# Serodynamics: A primer and synthetic review of methods for epidemiological inference using serological data

**DOI:** 10.1016/j.epidem.2024.100806

**Published:** 2024-12

**Authors:** James A. Hay, Isobel Routledge, Saki Takahashi

**Affiliations:** a Pandemic Sciences Institute, Nuffield Department of Medicine, University of Oxford, Oxford, United Kingdom; b Department of Medicine, University of California San Francisco, San Francisco, CA, USA; c Department of Epidemiology, Johns Hopkins Bloomberg School of Public Health, Baltimore, MD, USA

**Keywords:** Serology, Seroepidemiology, Serodynamics, Infectious disease modeling

## Abstract

We present a review and primer of methods to understand epidemiological dynamics and identify past exposures from serological data, referred to as serodynamics. We discuss processing and interpreting serological data prior to fitting serodynamical models, and review approaches for estimating epidemiological trends and past exposures, ranging from serocatalytic models applied to binary serostatus data, to more complex models incorporating quantitative antibody measurements and immunological understanding. Although these methods are seemingly disparate, we demonstrate how they are derived within a common mathematical framework. Finally, we discuss key areas for methodological development to improve scientific discovery and public health insights in seroepidemiology.

## Highlights

• There is a growing interest in using detailed, high-throughput immunological assays in infectious disease epidemiology
• We review current analytical methods for estimating epidemiological dynamics from serological data, termed serodynamics.
• We discuss key considerations for modelers, data requirements and limitations when modeling serological data.
• Despite different presentation, we show that most serodynamics models can be described by a single mathematical framework.

## 1 Introduction

Seroepidemiology is a key public health tool for understanding infectious disease dynamics and population health. The premise is to measure individual biomarkers, usually antibody levels or titers, from cross-sectional or longitudinal studies to infer epidemiological metrics such as the proportion of individuals who have previously been exposed to a pathogen or are immune to infection. Serological data are used widely to inform scientific understanding and improve public health decision-making by revealing incidence and prevalence trends, durability of immunity, and potential correlates of protection (Table 1). A particularly powerful aspect of serology is that a single cross-sectional sample can be used to estimate epidemiological dynamics over time, provided certain conditions are met (Hens et al., 2010). Developments in the throughput and detail afforded by modern serological assays alongside new analytical methods has led to an drastic increase in the generation of serological data in the past few decades, most recently in response to the SARS-CoV-2 pandemic (Arora et al., 2020).

**Table 1:** Key public health and research questions typically addressed using serological data.

Question	Description	Approach	Examples
What is an individual’s serostatus?	In the majority of seroepidemiological studies, serostatus is used to estimate attack rates, monitor progress towards elimination, and to estimate population susceptibility.	Choose a threshold antibody measurement or heuristic to classify individuals as seropositive/seroconverted or seronegative/seroreverted from one or more antibody measurements. The threshold depends on the assay, and is often pre-specified by the manufacturer or assay developer. The threshold may have biological meaning (e.g., the antibody titer associated with a 50% reduced risk of infection relative to a naive individual). Probabilistic classifications can also be made using mixture models.	(Cox et al., 2022, Bouman et al., 2021, Cauchemez et al., 2012, Chan et al., 2021)
What can we learn about immunological mechanisms from measured biomarkers?	Immune responses are highly dynamic and variable, but can be described using mathematical models. Serological data can be used to parameterize and predict the modeled kinetics of individual-level immunity and biomarkers.	Cohort studies, animal models and human challenge studies are used to sample (e.g., sera, plasma, saliva) individuals over time following infection or vaccination and measure serological markers such as antibody levels, T- and B-cell repertoires, and pathogen load. Key parameters such as immune waning or proportion of individuals who fail to mount an immune response can be parameterized using longitudinal study data, allowing biomarker kinetics and immune status to be predicted or projected in the wider population.	(Wei et al., 2021, White et al., 2019, McLaren and Potter, 1974, Huang et al., 2014, Antia et al., 2018)
Can serological data provide correlates of protection?	Serological measurements indicate pre-existing immunity and are correlated with the probability of infection or disease, and may explain more variation in outcomes than presence/absence of prior vaccination or infection. Correlates of protection are not well characterized for many pathogens.	Measure serological markers, usually antibody titers, in the population. Compare the rate of infection or disease between groups stratified by serological markers to estimate the relative risk of infection as a function of the marker. This estimate should either account for the time-varying force of infection in cohort studies or use controlled challenge studies.	(Tsang et al., 2014, Wei et al., 2022, Hobson et al., 1972, Plotkin, 2010, Alexandre et al., 2022, Anon, 2024)
What is the time-since-infection of a sample?	Antibody levels follow predictable trajectories following infection, and thus the timing of infection can be projected backwards from one or more antibody measurements.	Train a mathematical model to predict antibody level as a function of time-since-infection (TSI) or time-since-vaccination using longitudinal measurements following known exposure. For out-of-sample antibody titer or biomarker measurements with unobserved infection/vaccination timing, use the model to back-calculate when the individual was likely infected or vaccinated.	(Borremans et al., 2016, Rosado et al., 2021a, Simonsen et al., 2009, Sommen et al., 2011, Pelleau et al., 2021)
What were the historical epidemiological dynamics which led to the current serological landscape?	The distribution of antibody levels in the population at a given time reflects the convolution of past infection and vaccination dynamics in the population with within-host immunological kinetics.	Cross-sectional and/or longitudinal serosurvey data are used to estimate the TSI or seroconversion rate for many individuals. These individual TSI/seroconversion estimates are then combined to estimate the force of infection up to and/or within the sampling period using serocatalytic or time-since-infection models. These methods may also apply to reconstructing vaccination coverage.	(Zhao et al., 2018, Salje et al., 2021, Kucharski et al., 2018)
What is the population immune landscape for a particular pathogen?	The probability and size of an outbreak depends on both the transmissibility of an invading pathogen and the susceptibility of the population.	Serological studies can characterize the immune landscape of the population when paired with correlates of protection against the invading pathogen. For example, by measuring antibody titers effective against a particular pathogen across locations and age groups and generating an “average risk of infection” for each subgroup. Such studies require a) well-defined correlates of protection, and b) assays to measure susceptibility against the invading pathogen.	(Woudenberg et al., 2023, Fonville et al., 2014)

In this review, we provide an overview of analytical methods used to estimate past exposures and transmission dynamics from serological data, which we refer to as *serodynamics*. We describe key questions (Table 1), definitions (Table 2), and modeling considerations in seroepidemiology, highlighting key advances, and insights and challenges arising from new methods and data. We summarize principles common to many (but not all) pathogen systems, including discussion of the most appropriate models to address specific questions or to analyze particular types of serological data. We do not cover all uses for serology in public health (Metcalf et al., 2016, Cutts and Hanson, 2016, Wiens et al., 2022, Haselbeck et al., 2022), wildlife ecology (Pepin et al., 2017, Gilbert et al., 2013), and immunological modeling as these topics have been comprehensively reviewed by others (Handel et al., 2019, Garcia-Fogeda et al., 2023). Similarly, the choice of study design for seroepidemiology has been reviewed recently and is not covered here (Clapham et al., 2020, Pan American Health Organization, 2022).

**Table 2:** Glossary of key terms used in seroepidemiology and serodynamics.

Term	Definition
Antibody binding	Refers to binding of antibodies to an antigen. For example, ELISAs (binding serological assays commonly used in infectious disease serology) detect and quantify antibodies bound to specific antigens attached to a plate. Binding antibody titers are typically morphological measurements (e.g., optical density in an ELISA reader) in contrast to *antibody neutralization* measurements.
Antibody class	Classification of antibodies (i.e., immunoglobulins (Ig)) with differing properties and functions. There are 5 antibody classes (also referred to as isotypes) in humans: IgG, IgM, IgA, IgD, IgE. In infectious disease serology, the most frequently tested antibody class is IgG (long-term markers of prior exposure), followed by IgM (short-term markers of recent exposure) and IgA (mucosal responses).
Antibody kinetics	The values of/changes in the titer or level of antibody over time, within an individual, following an immune exposure (e.g., rapid boost followed by more gradual decay). Often used interchangeably with *antibody dynamics* in the literature, though in this review we use *antibody kinetics* to refer to the within-host level and *antibody dynamics* to refer to the population level.
Antibody neutralization	Refers to ability and amount of antibody required to neutralize infection (measured by functional serological assays), and are typically considered to be the gold standard for infectious disease serology. Neutralization antibody units are in titers.
Antibody titer	The inverse of the highest dilution (lowest concentration) of a sample that still gives a positive serological result, typically on a neutralization assay. For this review, analytical methods are applicable to both antibody titers and morphological measurements (e.g., optical density or fluorescence intensity), and we use “antibody titer” to broadly mean “quantitative antibody response”.
Antigen	A molecular structure encountered by, presented to, and recognized by the immune system which triggers an immune response (and for the purposes of this review, antibody production).
Antigenic variation	Pathogens are antigenically variable when the antigens targeted by a host’s immune system can undergo structural change, allowing them to avoid pre-existing immunity and cause reinfection.
Binary (serological) data	Serological data that are dichotomized into a seronegative or seropositive result.
Biomarker	A broad term representing an analyte of interest (e.g., a biomarker of interest could be the titer of antibody class *X* which binds to antigen *Y* from pathogen *Z*).
Epitope	The molecular region on the surface of an antigen that is recognized by the immune system and bound by an antibody molecule (or T- or B-cell).
Exposure	An event of the immune system encountering an antigen leading to the production of an antibody response. For the purposes of this review, an “infectious disease exposure” or “immune exposure” (used interchangeably) refers to infection, re-infection or vaccination.
Force of infection (FOI)	The per capita rate at which susceptibles are exposed to infection. The FOI (*λ*) is also the hazard of infection from the survival analysis literature. The units are 1/time (typically 1/year).
Immune history	An individual’s set of previous exposure events, usually defined in terms of the timing, antigens and route of exposure (e.g., infection or vaccination).
Immune landscape	The level of immunity or susceptibility to an infection or disease across a population, where “landscape” may refer to geographical location, demography, antigenic space etc.
Immune profile	An individual’s level of immunity or susceptibility to a particular infection or disease, often described by the presence or quantity of one or more biomarkers. Sometimes referred to as an *antibody profile* or *antibody landscape* when using antibodies as a proxy for immunity.
Quantitative (serological) data	Serological data that preserve/use the quantitative result from the assay and correlate with the amount of antibody present. Common units include fluorescent intensity or titer.
Seroconversion	The change in an individual’s immune status from seronegative to seropositive (e.g., due to an immune exposure). This term can also be used at the population level (i.e., seroconversion rate is sometimes used interchangeably with seroincidence rate in the literature).
Seroincidence	The population-level incidence (i.e., occurrence) of detectable immune exposures (i.e., infections) over a specific time period. Seroincidence can be quantified as a proportion (percentage of a population that was exposed) or as a rate (exposures per person-time at risk).
Serological landscape	Similar to an immune landscape, describing the distribution of seropositivity or antibody levels across a population.
Seroprevalence	The proportion of a population that is seropositive for a given biomarker. Seroprevalence is often used as a proxy for cumulative incidence or the proportion of the population who are immune.
Seroreversion	The change in an individual’s immune status from seropositive to seronegative (e.g., due to antibody waning). This term can also be used at the population level (i.e., seroreversion rate is used to describe the rate of seroreversions in a population over time).
Serostatus	The binary classification of an individual with respect to a given biomarker, seropositive or seronegative.
Time-since-infection (TSI)	The time between an exposure (usually infection, but sometimes vaccination) event and the sampling time. A TSI model describes the relationship between the TSI and antibody level at the individual level.

Section 2 provides a primer on sources of serological data and considerations for data pre-processing and interpretation prior to fitting serodynamical models. Section 3 reviews methods using binary serostatus data to reconstruct transmission dynamics. Section 4 covers recently developed methods using (semi-)quantitative serological data to reconstruct antibody kinetics, transmission dynamics and infection histories (also referred to as *time-since-infection* methods). The Appendix provides a detailed derivation of these serodynamics methods within a common mathematical framework, highlighting their common foundation in survival analyses and linking to the classic relationship between incidence and prevalence. Section 5 closes with a discussion of key research directions and opportunities in serodynamics.

## 2 Sources and pre-processing of serological data

This section summarizes key considerations when analyzing serological data, from the point of sample collection through to generation of a single measurement. We first describe common serological study designs and assays used to generate raw data. Analysts and modelers will often use only a final, summary measurement per sample (e.g., the dilution factor corresponding to some pre-specified signal threshold), but we discuss common sources of variation and error introduced during the laboratory process which should be considered in downstream analyses. Finally, we discuss key considerations and caveats when converting raw assay output to binary serostatus.

### 2.1 Study designs commonly used in seroepidemiology

Serological data are most often collected following two classic epidemiological study designs: a *cross-sectional survey* or a longitudinal *cohort study* (Clapham et al., 2020, Horby et al., 2017). Other sources of serological data include controlled human challenge experiments (Barclay et al., 1989, Achan et al., 2020) and clinical trials (Lipsitch et al., 2020). When serology is performed as part of public health surveillance this is often referred to as *serosurveillance*. While the bulk of seroepidemiological studies have been on analyzing antibodies in the blood, the use of alternative media, particularly oral fluid (Heaney et al., 2021), has immense public health potential for uptake and scalability. However, the convenience of these alternative sample types is often traded-off against other important properties such as storage, ease of use, and reduced sensitivity and specificity.

In a cross-sectional serological survey (*serosurvey*), specimens from individuals in a defined population are tested for antibodies (typically IgG responses) against a pathogen. *Seroprevalence* is calculated as the proportion of the population that tested positive for antibodies (seropositive). Cross-sectional serosurveys can be conducted at either a single time-point or serially over time in the population, and conducted using either probabilistic (e.g., (Salje et al., 2019)) or non-probabilistic, convenience-based sampling methods. Examples of the latter include sampling of residual specimens from blood banks, health facilities, and commercial laboratories, all of which became more commonly performed as part of the response to the global SARS-CoV-2 pandemic (Arora et al., 2020).

Longitudinal cohort studies follow the same individuals over time and collect serological data at multiple time-points (e.g., (Arnold et al., 2019)). In addition to seroprevalence, cohort studies can be used to estimate *seroincidence* (the proportion or rate of individuals who are infected during a specified time interval) and other parameters such as the proportion of infections that are asymptomatic or subclinical. When paired with known dates of infection or vaccination these data sets provide an important source of information for parametrizing the magnitude and kinetics of biomarkers after exposure (see Section 5). Furthermore, serological data can be collected in household transmission studies where the entire household of an index case is enrolled and followed to estimate parameters such as the secondary attack rate and to test the effectiveness of interventions (e.g., (Jackson et al., 2011)). Lastly, serological data can be generated through other use cases, including clinical trials for stratification of baseline risk or as a surrogate outcome (Lipsitch et al., 2020).

### 2.2 Laboratory assays commonly used in seroepidemiology

The generation and interpretation of raw serological assay output varies across common assays. Table 3 describes the types of serological assays typically used in seroepidemiological studies. Except for the functional neutralization assays (which measure the amount of antibody that can potentially neutralize infection and are usually considered to be the gold standard), these assays are typically binding assays which detect and quantify the amount of binding of the antibody to the antigen, which may or may not be immunologically relevant. Assays are also used to evaluate binding avidity (i.e., the overall strength of interaction between an antigen complex and antibody; not to be confused with binding affinity, the strength of interaction between an antigenic epitope and antibody binding site) (Garcia et al., 2022, Ssewanyana et al., 2020). Functional assays are much more resource-intensive to perform than binding assays, and binding assays are often used for seroepidemiological applications as binary and quantitative measurements tend to be well-correlated between the two assay types (Lutz et al., 2023, Suhandynata et al., 2021, Brown et al., 2014).

**Table 3:** Serological assays commonly used for serosurveillance and research.

**Assay type**	**Features**	**Signal units and what is being measured**	**Number and biological scale of analytes typically screened**	**Considerations for data analysis and interpretation**
**Serological assays commonly used for serosurveillance and research**				
**Enzyme immunoassays (EIA, ELISA)**	Commercial and in-house research use assays available for many pathogens.	Binding assay Units: optical density (OD), a morphological measurement of binding	1 antigen (whole protein or sub-domain of interest)	Produces binary serostatus (enzyme immunoassays) or continuous measurements. Enzyme immunoassays typically have limited dynamic range, compared to other assays. ODs and FIs can be converted to standardized units comparable to an antibody concentration or titer, using serial dilutions of a monoclonal antibody or positive control.
**Multiplex bead assays (MBA)** Also referred to by manufacturer or platform name, e.g., Luminex or xMAP®	Can be multiplexed (simultaneous detection of responses to multiple antigens). Panels available for many pathogens from research groups (e.g., SARS-CoV−2 (Pelleau et al., 2021), vaccine-preventable diseases (Smits et al., 2012), neglected tropical diseases (Arnold et al., 2017), malaria (Wu et al., 2019)).	Binding assay Units: fluorescence intensity (FI), a morphological measurement of binding	1–10 s of antigens (whole proteins or sub-domains of interest)	
**(Hem)agglutination (inhibition) assays (HI/HAI)**	Conventionally used for viruses with a hemagglutinin protein (e.g., influenza, measles).	Functional assay (i.e., antibody preventing agglutination of red blood cells) Units: titer	1 antigen (whole protein)	Produces discretized data (typically, reciprocal of the highest dilution of sera for which pathogen replication has been reduced by 50%; known as inhibitory concentrations 50% (IC50)). Titers from functional assays are typically a better proxy of the immunological quantity of interest (i.e., immunity) than metrics from binding assays.
**Neutralization assays**	Considered the gold standard serological assay. Labor- and resource-intensive to perform (on the order of days). Examples include: plaque reduction neutralization tests (PRNTs); micro-neutralization tests; pseudotyped virus neutralization tests; bactericidal tests.	Functional assay (i.e., *in vitro* antibody neutralizing infectivity by binding to the antigen and preventing cytopathic infection) Units: titer	1 antigen (whole pathogen)	
**Serological assays used for research**				
**Protein microarrays**	Can detect multiple antibody classes/isotypes simultaneously. Examples from infectious disease applications: malaria (Helb et al., 2015); plague (Keasey et al., 2009).	Binding assay Units: FI	>100 s of antigens (whole proteins)	Bioinformatic approaches for pre-processing and normalization are under active development. Biological interpretations of raw assay results are not readily available or straightforward. Data-generating process is too complex for standard modeling approaches, so typically framed as (unsupervised or supervised) machine learning questions.
**Phage display** Also referred to as phage immunoprecipitation sequencing (PhIP-Seq)	Specific libraries include: VirScan (all known human viruses) (Xu et al., 2015); human coronaviruses including SARS-CoV−2 (Morgenlander et al., 2021); malaria (Raghavan et al., 2023).	Binding assay Units: sequencing reads counts	>1000 s of antigens (linear epitopes)	

Recent technological advances have made it possible for multiplexing (e.g., using multiplex bead assays), which allows for the simultaneous detection of dozens of antibody responses to multiple antigens in the same experiment. These multisera data enable a cost-effective approach to serosurveillance, enabling identification of sub-populations with high exposure to multiple pathogens (Fornace et al., 2022), more accurate monitoring of intervention effectiveness (Parameswaran et al., 2021), and increased specificity to estimate exposure history for pathogens exhibiting cross-reactivity (Hozé et al., 2020). Further, comparisons of antibody responses measured in monoplex and multiplex are needed to ensure that measured signals are not affected by assay plexity. Additional state-of-the-art research assays, such as protein microarrays and phage display, have enabled simultaneous detection of hundreds to thousands of antibody responses; bioinformatic methods development to obtain biological and epidemiological signals from these high-dimensional data is an active area of current research (Monaco et al., 2021, Bérubé et al., 2023).

### 2.3 Sources of variation in the measurement process

**Fig. 1:**
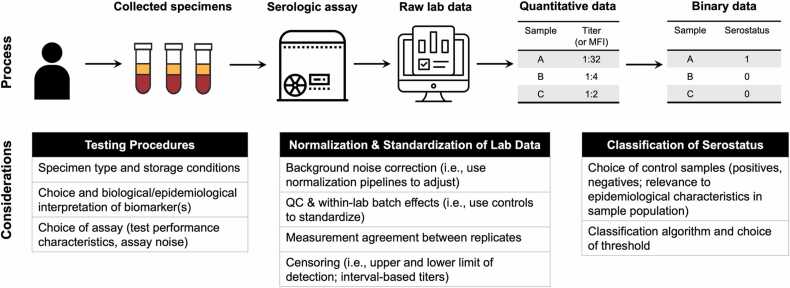
From blood vials to processed seroepidemiological data and sources of variation in the observation process. Tables include upstream considerations in the measurement process (i.e., testing procedures, normalization and standardization of lab data, and classification of serostatus) that can affect downstream serodynamics applications.

Given the range of biomarkers and assays, one of the biggest challenges for interpreting and modeling serological data is to differentiate between epidemiologically meaningful variation, which might reflect differences in immunity or transmission, from noise (technical variation) introduced during the sample processing pipeline (Fig. 1). For example, the same sample run on the same assay at different labs or between experimental batches can lead to different results (Wood et al., 2012); repeated freeze-thaw or poor storage conditions might lead to sample degradation (Torelli et al., 2021); and different strains or stocks of the same antigen might perform differently in an otherwise identical assay (Li et al., 2013, Salje et al., 2018).

Laboratory pipelines and protocols are generally designed to standardize and minimize the impact of these sources of variation (Windsor et al., 2022, Mattiuzzo et al., 2019, Zacour et al., 2016). Other key steps include quality control (QC), adjusting for background noise and batch effects, including positive controls, relating observed measurements to a standard curve from a dilution series, and assessing measurement agreement between technical replicates (Fig. 1). Examples of bioinformatic pipelines for processing raw serological data include (covidClassifyR, 2023; flexfit, 2023).

In modeling terms, the parameter of interest is the true biomarker level in the individual at the time of sampling which must be inferred conditional on the observed measurement after accounting for the sources of noise and bias outlined in Fig. 1. For binary serostatus data, this variation accounts for imperfect assay sensitivity and specificity, where the true serostatus or seroprevalence is a latent binary variable to be estimated (Larremore et al., 2021, Zhang et al., 2013). For quantitative biomarker data, observed antibody titers are often assumed to be randomly distributed around some true, unknown value. More complex data types, such as those arising from protein microarray data or multiplex assays require more complex hierarchical models to account for multiple sources of variation and normalization in the observation process (Bérubé et al., 2023). Overall, sources of variation in the laboratory pipeline can in theory be modeled to produce corrected antibody measurements for each sample (Gelman et al., 2004), but it is often easier (and necessary) to adjust for these random and systematic biases at the experimental design phase.

### 2.4 Considerations when converting raw laboratory measurements into binary serostatus

Although raw laboratory measurements are often quantitative, serological data are most widely reported as binary, in part due to the challenge of distinguishing between noise and signal from (semi-)quantitative measurements. Individuals are classified as either *seropositive* or *seronegative* as a result of processed antibody measurements being above or below a given threshold at the time of sampling, or as having *seroconverted* as a result of increases in antibody levels above a predefined threshold between two timepoints.

There are at least four common ways to define a threshold to convert quantitative measurements into a binary serostatus (Chan et al., 2021). First, on a commercial serological assay, the cutoff specified by the assay manufacturer can be used to determine binary serostatus (often, there will be a third category of ‘indeterminate’ or ‘equivocal’ for samples demonstrating borderline reactivity). Second, if the researcher has access to positive and negative controls (samples from individuals known to have had prior exposure and individuals known to not have had prior exposure), then a cutoff can be determined using a ROC curve that maximizes the test performance characteristics of interest (sensitivity, specificity, or both). Third, if the researcher has access to negative controls only (e.g., for recently emerged infections), the cutoff can be informed by the distribution of responses in these samples (typically using a cutoff of 2 or 3 standard deviations above the mean). Fourth, if a correlate of protection exists (e.g., for measles (Bolotin et al., 2019)), then this value can be used as the cutoff, and the binary serostatus would thus reflect binary seroprotection status.

Probabilistic modeling approaches can be used instead to account for classification uncertainty. One approach used, albeit less frequently, is to fit a finite mixture model to distributions of quantitative serological results. Mixture models assume that samples are taken from a mixture of two (or more) distributions with parameters representing different latent subpopulations: one population which has previously experienced infection or vaccination and one population which has not, where the relative weighting of these two components in the overall distribution gives the seroprevalence (McLachlan et al., 2019, Hardelid et al., 2008, Gay et al., 2003) (Appendix 2.1). These approaches are appropriate when distributions of quantitative antibody response distributions are distinct between latent subpopulations.

### 2.5 Caveats in interpreting binary serostatus data

There are several challenges in generating and interpreting binary serostatus data, arising during the measurement process (Fig. 1) and through genuine biological variation (Table 5). Serological assays vary greatly in their sensitivity and specificity, and existing assays and corresponding cut-points may have been optimized to detect different outcomes. This can be a particular challenge for defining seroconversion, where sub-threshold antibody boosts may be missed due to measurement errors or by setting the seropositivity/seroconversion threshold too high (Cauchemez et al., 2012, Zhao et al., 2017, Quandelacy et al., 2021). True biological variation, such as antibody waning over time, can also present a challenge. For example, taking a serum sample too early after vaccination may capture an individual before their antibody response has had time to peak (Wei et al., 2021, Miller et al., 2010, Veguilla et al., 2011). Censoring effects due to the discrete nature of some assays, antibody activity below the threshold for detection, and antibody boosting near the upper dilution limit may also limit the ability to identify seroconversions.

Finally, it is important to consider the antigen target of the assay with respect to the outcome of interest. For example, an individual might mount a substantial immune response following infection or vaccination directed primarily against an antigen which is not measured by the assay (Monto et al., 2015). Cross-reactivity within or between pathogen species can also make it difficult to interpret serostatus data – antibodies generated in response to exposure to one variant may also bind to antigenically related variants by targeting conserved epitopes, with the degree of cross-reactivity dependent on how antigenically similar two variants or pathogens are (Priyamvada et al., 2016, O’Driscoll et al., 2024).

## 3 Methods using binary serostatus data to estimate epidemiological parameters and reconstruct transmission dynamics

Serological data are commonly used in epidemiological analysis as binary variables, measuring whether an individual is seropositive or seronegative. In this section, we explain how binary serostatus data are used to estimates key epidemiological parameters such as seroprevalence, infection ascertainment rates, and infection-fatality ratios. We then describe the classic serocatalytic model used to estimate the force of infection from age- or time-stratified seroprevalence data, focusing on necessary assumptions, requirements and limitations.

### 3.1 Using binary serostatus data to estimate seroprevalence and epidemiological parameters

Binary serological data can be used to estimate several key epidemiological metrics. One such metric is the *cumulative incidence* (the total number of new cases in a population over a period of time, also referred to as the *attack rate*; Appendix 1.1). For pathogens where the antibody response is long-lived or where the antibody response has not yet waned (for example, following a recent outbreak in a naïve population), cumulative incidence can be simply calculated as the proportion of the population which is seropositive (or seroconverted), although due to the time taken to mount an immune response, results may be lagged by a few weeks. Estimates of cumulative incidence that adjust for test performance characteristics (e.g., imperfect sensitivity due to non-responders; imperfect specificity due to cross-reactivity) can be produced using the Rogan-Gladen estimator (Rogan and Gladen, 1978) or binomial models for sensitivity, specificity, and cumulative incidence (Larremore et al., 2021). If the antibody response is not long-lived or has begun to wane, adjustments for seroreversion (Brazeau et al., 2022, Loesche et al., 2022) must be made (Appendix 1.2).

Many other programmatically useful metrics, such as ascertainment rates and infection-fatality ratios are derived from seroprevalence, depending on the transmission context and epidemiology of the pathogen of interest. Vaccination coverage and the proportion of the population immune to disease are also key metrics obtained through serosurvey data, which help to monitor the impact of interventions and progress towards disease control and elimination goals for endemic pathogens (Wiens et al., 2022, Pan American Health Organization, 2022, Sepúlveda et al., 2015).

### 3.2 Using serocatalytic models to estimate FOI

**Fig. 2:**
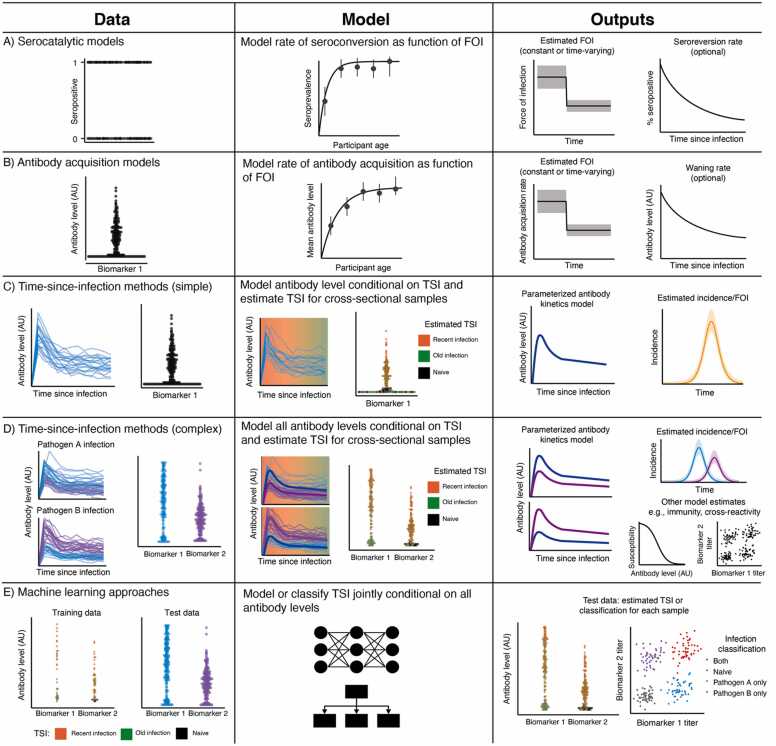
Overview of serodynamics methods for reconstructing transmission dynamics. Different serodynamics methods can be used to estimate transmission dynamics depending on the type and availability of data. For each approach (row), the figure shows data requirements, a depiction of the model structure, and key model outputs. (A) Serocatalytic models require only binary serostatus and age to link age-stratified seroprevalence to the FOI, optionally accounting for additional complexity such as seroreversion. (B) Antibody acquisition models have a similar structure to serocatalytic models, but using antibody level rather than serostatus. (C) Simple TSI methods use quantitative antibody levels combined with a model of expected antibody kinetics to back-calculate an individual’s likely infection time from one or more measurements. This simple approach can be extended to account for co-circulation, reinfection and cross-reactivity of multiple pathogens and measurement of multiple biomarkers, enabling fitting of more complex models (D). (E) Machine learning approaches rely on similar input data and may be used to answer similar questions regarding TSI, but do not explicitly model the data-generating process.

For pathogens that typically cause infrequent infections followed by durable or well-characterized immune responses, the per capita rate of new infections–the force of infection (FOI)–can be estimated by fitting a serocatalytic model to age-stratified seroprevalence data (Fig. 2A; referred to as *age-based* methods in Pepin et al. (2017); (Hens et al., 2010, Hens et al., 2012). Serocatalytic models are based on the intuition that individuals are typically infected earlier in life when subject to a high FOI compared to a low FOI, and thus the average age of seroconversion and the age-distribution of seropositivity can be used to estimate the FOI (Appendix 1.3). These relatively simple models, combined with inexpensive and simple antibody assays, continue to be widely used for epidemiological analysis. Note that the FOI estimated using the serocatalytic model can be used to derive other key epidemiological parameters such as the basic reproduction number, though this can be challenging in practice due to non-identifiability between age-stratified infection rates (measured by serology) and age-stratified infectivity, which requires additional data on population behavior (Wallinga et al., 2006, Farrington et al., 2001, Ferguson et al., 1999).

A key assumption of the basic serocatalytic model is that the FOI is constant for all ages and over time. However, this assumption has been relaxed in many studies to estimate a time- and/or age-varying FOI (e.g., due to the introduction of interventions, or seasonal dynamics). Most of the complexity in implementing a time-varying FOI model comes down to the way in which the FOI is modeled over time. Early implementations of serocatalytic models with time-varying FOI used parametric methods, for example by assuming the FOI is a linear polynomial function of time (Grenfell and Anderson, 1985, Griffiths, 1974, Farrington, 1990). An alternative approach is to treat the FOI as a vector of parameters corresponding to different time periods, where the individual FOI parameters are assumed to be conditionally independent given some model structure or prior (e.g., using smoothing splines or a random walk prior) (Keiding, 1991, Cucunubá et al., 2024). Treating the FOI as a piecewise constant is also common, allowing a model to estimate the FOI before and after some anticipated change in transmission intensity (Nakajo and Nishiura, 2023). The choice between discrete and continuous time, and the choice of model structure for the time-varying FOI depends on the informativeness of the data, the immunology and epidemiology of the pathogen, the hypothesis being tested, and practical considerations of model fitting; more complex models require more data and computation to enable parameter identifiability.

Another assumption of the basic serocatalytic model is that individuals remain seropositive indefinitely. Methods have been developed to relax this assumption for pathogens where immunity wanes over time, and thus where seroprevalence does not necessarily increase monotonically with age. These so-called *reversible serocatalytic models* include a parameter to describe the rate of seroreversion. Reversible serocatalytic models are useful both where the seroreversion rate itself is of interest, and where failing to account for seroreversion can bias estimates of the FOI. For example, (Rees et al., 2022) fit a reversible serocatalytic model to Leptospirosis serological survey data collected in Fiji to jointly estimate the time-varying FOI, duration of antibody positivity, and most likely timing of individual infections. Reversible serocatalytic models have also been used to estimate seroconversion and reversion events in longitudinal serosurveys for malaria (Simmons et al., 2017, Wu et al., 2020, Yao et al., 2022).

Finally, serocatalytic models have been extended to multi-strain pathogens, such as dengue, where multiple serotypes may co-circulate and strains may interact via the host immune response. In this case, strain-specific FOIs and in turn the basic reproductive number *R*_*0*_, can be estimated from cross-sectional age-stratified serological data, though this usually requires the simplifying assumptions of homogenous mass-action mixing and an age-independent FOI (Ferguson et al., 1999, Rodríguez-Barraquer et al., 2014).

### 3.3 Requirements and limitations of the serocatalytic model

Data requirements for the serocatalytic model are fairly minimal, though more complex models require additional variables, summarized in Table 4. These variables are not always reliable nor obtainable, for example, estimating individual ages in wildlife populations (Gilbert et al., 2013). In these instances, longitudinal or repeated cross-sectional samples are required to provide information on how seroprevalence may have changed over time (Figure S3). In longitudinal studies following the same individuals over time, the FOI can be estimated directly as the number of seroconversions per person-time at risk (Arnold et al., 2019). Antibody kinetics can also add complexity, particularly when antibody waning rates are not well characterized, making it challenging to distinguish between naive individuals and those who have seroreverted in a cross-sectional serosurvey. Furthermore, unless appropriate study designs and biomarkers are chosen, models may not be able to distinguish infection from other causes of seroconversion such as cross-reactivity and vaccination.

**Table 4:** Data considerations for serocatalytic models, depending on use case.

**Metric to estimate**	**Minimum data needed**	**Use cases**	**Examples**
Population-wide FOI	Proportion seropositive by age	Estimating ascertainment rates, population-level seroprevalence, average age at first infection	(Cucunubá et al., 2017, Salje et al., 2016, Bailly et al., 2022)
Age-specific FOI	Proportion seropositive by age	Estimating timing of historical outbreaks	(Bailly et al., 2022)
Time-varying FOI	Proportion seropositive by age	Estimating timing of historical outbreaks, impact of interventions, changes in transmission rates over time	(Cucunubá et al., 2017, Salje et al., 2016)
FOI in different populations	Proportion seropositive by age, time and population	Estimating impact of interventions on different populations and over time. Identifying health inequities or risk factors.	(Cucunubá et al., 2017, Ribeiro Dos Santos et al., 2022)
FOI with seroreversion	Proportion seropositive by age and/or time, seroreversion rate (known or estimated from data)	Estimating the force of infection and duration of seropositivity in cases where immunity wanes and is not lifelong	(Rees et al., 2022, Simmons et al., 2017, Wu et al., 2020, Pinsent et al., 2018)

A final consideration is how to link the model to the data and estimate the model parameters. It is common to use a binomial likelihood function, where the serocatalytic model generates expected seroprevalence per age group and the data are counts of seropositive and seronegative individuals. However, the use of binary serostatus data brings the same limitations of discretizing antibody responses discussed in Section 2.4, such as choice of seropositivity threshold, imperfect assay accuracy, and indeterminate classifications which are often discarded. Fixing serostatus before fitting a model risks underestimating uncertainty in the inferred epidemiological parameters such as FOI or cumulative incidence, as this fails to incorporate uncertainty in the data classification. One solution is to use more complex likelihood functions which do not require serostatus to be assigned prior to fitting the model. For example, Bollaerts et al. developed a method to fit a serocatalytic model to *Salmonella* and Varicella-Zoster virus antibody titer data directly by embedding a two-component mixture model, where one component of the mixture represents infected and the other uninfected individuals (Bollaerts et al., 2012).

## 4 Methods using quantitative or semi-quantitative antibody measurements to reconstruct epidemiological dynamics

The methods discussed so far rely on the binary categorization of seronegative and seropositive individuals (Fig. 2A). However, binarizing (or categorizing) serological data can lead to misclassification (Cauchemez et al., 2012, Chan et al., 2021) and losing information encoded in the magnitude of the serological response (Bollaerts et al., 2012, Fedorov et al., 2009). An alternative class of methods, referred to as *quantitative antibody methods,* can yield greater insights by using the antibody measurement rather than a binary serostatus (Pepin et al., 2017). An underappreciated point is that the mathematical process describing the generation of binary serological data is the same as that for quantitative data – observed serological measurements at a given point in time reflect the convolution of incidence, which determines the distribution of times-since-infection in the sample, and the within-host level, which determines the distribution of measurements for a given time-since-infection (Figure S2). Whether these measurements are binary or quantitative is determined by how the biomarker or antibody level is measured (i.e., choice of assay), or a result of binarizing continuous data prior to analysis. This commonality is shown in the Appendix. In this section, we describe the key components of models for estimating the FOI and individual infection histories using quantitative serological data. An active area of research is the extension of quantitative antibody methods to multi-pathogen systems, which brings a number of additional challenges discussed here Table 5.

**Table 5:** Sources of biological variation impacting serological data.

**Source of variation**	**Description**	**Approach**	**Examples**
Pathogen factors	• Different pathogens and host immunological responses exhibit different within-host kinetics (e.g., measles vs. tetanus antibody waning rates (Antia et al., 2018, Amanna et al., 2007, Hodgson et al., 2024); response to acute vs. chronic infections).	• Estimate parameters for the pathogen of interest using longitudinal data with known time of infection or onset. • Kinetics may be more similar within than between species (e.g., different SARS-CoV−2 variants or influenza subtypes), which can form the basis for priors.	(Antia et al., 2018, Amanna et al., 2007, Freeman et al., 2016)
Exposure type and dose	• The composition and dose of exposure to an antigen determines the immunological response e.g., which antigens are included in a vaccine. • The route of exposure determines the location of antigenic stimulation (e.g., an intranasal vaccination may trigger a strong mucosal response whereas an intramuscular vaccination may not).	• Antibody levels against a subset of possible antigens are stimulated depending on the antigenic composition of the exposure. • Longitudinal data on post-exposure kinetics are needed for each exposure type being modeled.	(Fonville et al., 2014, Kang et al., 2013, Markewitz et al., 2022, Berbers et al., 2013)
Host factors	• Host factors such as age, sex, comorbidities, coinfection and being immunocompromised can affect biomarker kinetics (Scepanovic et al., 2018, Haq and McElhaney, 2014). • Genetic factors such as human leukocyte antigen (HLA) class have also been shown to explain variation in humoral immunity (Scepanovic et al., 2018, Graham et al., 2010).	• Stratifying kinetics model parameters into demographic groups or using hierarchical models with sufficient data. • If subgroup differences are small, they may not substantially affect estimates, or might be infeasible to model, and thus might be ignored.	(Ranjeva et al., 2018, Scepanovic et al., 2018, Graham et al., 2010)
Individual-level variation	• Within-host kinetics are stochastic such that two identical exposure events might lead to different biomarker kinetics. • Between-individual variation in kinetics may be greater than can be explained by measurable host factors.	• Use random-effects terms on individual-level within-host kinetics parameters. • This can dramatically increase the number of parameters to be estimated, and is thus best suited to rich datasets with multiple observations per individual.	(White et al., 2019, Zhao et al., 2018, Kucharski et al., 2018, Salje et al., 2018, Hay et al., 2020, Kucharski et al., 2015a, Graham et al., 2010)
Infection history	• Within-host kinetics depend on pre-existing immunity e.g., IgG titers are boosted more quickly upon re-exposure, whereas IgM titers are only elevated following primary exposure. • Antibody responses following reinfection involve both *de novo* plasmablasts and memory B cells targeting multiple epitopes, which can lead to effects such as immune imprinting, titer-ceiling effects and antigenic seniority.	• Ideally, each individual’s infection history would be known or jointly estimated alongside other parameters, though this is usually infeasible without considerable data. • Simplifications may be made such as using random-effects terms to allow variation between each modeled exposure, or to model kinetics as a function of baseline biomarker levels.	(White et al., 2019, Zhao et al., 2018, Kucharski et al., 2018, Salje et al., 2018, Hay et al., 2020, Kucharski et al., 2015a, Tsang et al., 2019)
Target biomarker	• Antibodies to different antigens, different immunoglobulins, and different types of biomarker (e.g., avidity) exhibit different degrees of boosting and waning. • This can provide orthogonal information on time-since-infection, but is also a potential pitfall when comparing kinetics parameters using different assays or targets.	• Use separate or multi-level kinetics models to jointly describe kinetics to multiple biomarkers without assuming shared parameter values. • Model only one or a subset of most informative biomarkers from all those measured.	(Pelleau et al., 2021, Arnold et al., 2017, Tsang et al., 2019, Jones et al., 2022, Rosado et al., 2021b, Wiens et al., 2023, Radtke et al., 2021)

### 4.1 Antibody acquisition models

An extension of the serocatalytic model is to use individual-level antibody measurements in different age groups as the outcome variable instead of seroprevalence (Bollaerts et al., 2012, Lam et al., 2019). Fitting serocatalytic models to quantitative antibody data using mixture models (Section 2.4) is one way in which these full data can be used; however, these models still ultimately estimate a binary serostatus for each individual (Bollaerts et al., 2012, Del Fava et al., 2016). An alternative group of models, termed *antibody acquisition* or *antibody density* models (Fig. 2B), assume instead that antibody levels increase over time proportional to the force of infection without assigning serostatus using the differential equation described in (Yman et al., 2016).

Antibody acquisition models have strengths and weaknesses compared to serocatalytic models depending on the epidemiological context. Acquisition models are mainly used in pathogen systems where individuals experience multiple serological boosts over their life, and thus seroprevalence becomes saturated over time, particularly when antibody decay rates are low. Measuring the transmission intensity of malaria is a key example: infection does not lead to sterilizing immunity, individuals are infected many times over their life, and the duration of seropositivity varies between antigens (Yman et al., 2016, Weber et al., 2017, Pothin et al., 2016). Antibody acquisition models also provide the ability to estimate epidemiological trends with typically greater power and precision than serocatalytic models due to the use of quantitative antibody measurements (Yman et al., 2016). However, these models can be difficult to interpret, as they do not estimate the force of infection directly but rather a proxy in the form of antibody acquisition rates. Furthermore, it can be challenging to compare antibody acquisition rate estimates from different assay platforms where antibody levels may not be comparable, as demonstrated by Pinsent et al. in their application of antibody acquisition models to estimate the transmission intensity of trachoma (Pinsent et al., 2018).

### 4.2 Time-since-infection methods for single exposures

An alternative approach to estimating epidemiological trends from quantitative serological data exploits the fact that individual-level antibody levels, or biomarkers more broadly, change predictably over time following exposure. When the within-host processes governing changes in biomarker levels over time-since-infection (TSI) or time-since-vaccination are well characterized, the timing of an individual’s infection or vaccination can be back-calculated conditional on their observed biomarker levels from cross-sectional or longitudinal samples (Boni et al., 2019) (Figure S4D). Individual infection time estimates can then be combined to estimate population-level trends. These multi-level modeling approaches are usually referred to as time-since-infection methods, titer-based methods (Pepin et al., 2017), or sometimes age-of-infection methods, with links to foundational work in (Kermack and McKendrick, 1991, Brauer, 2005) (Fig. 2C).

#### 4.2.1 The within-host level: models for biomarker kinetics

A key component of all time-since-infection methods is to explicitly model the kinetics of biomarker levels over time after infection or vaccination (Pepin et al., 2017, Borremans et al., 2016, Simonsen et al., 2009, Hay et al., 2020, Rydevik et al., 2016) (Figure S1). Most implementations of these within-host models can be thought of as a form of generalized linear modeling: biomarker levels are random variables distributed according to a model describing their expectation and distribution at each possible TSI (Figure S4B).

Building and parameterizing a mechanistic model describing the full set of immunological processes which determine post-infection biomarker kinetics is challenging, and within-host modeling is an active area of research in its own right (reviewed in (Garcia-Fogeda et al., 2023)). There are many mathematical modeling studies which focus on fitting mechanistic models of immunological processes to multiple biomarkers measured following exposure to a pathogen in controlled conditions (e.g., challenge studies) to understand different arms of the immune response. This has uses for quantifying the effect of therapeutics such as antivirals (Canini et al., 2014), understanding the timing and contribution of different arms of the immune response (Anon, 2017), projecting long-term serostatus (Clairon et al., 2023), as well as potentially identifying mechanisms of protection (Alexandre et al., 2022).

In contrast, most practical within-host models used for seroepidemiological inference focus on prediction or classification of infection recency rather than capturing underlying mechanisms. For example, the first TSI estimation methods developed to estimate human immunodeficiency virus (HIV) incidence from cross-sectional sera classified infections as within a window period soon after infection or long into their infection, based on measured anti-HIV IgG titers and avidities (Sommen et al., 2011, Brookmeyer and Quinn, 1995, Murphy and Parry, 2008, Brookmeyer, 2010, Le Vu et al., 2008). More complex descriptions of biomarker kinetics include power-law models based on the Lotka-Volterra model (Teunis et al., 2012, de Graaf et al., 2014, Teunis et al., 2016, Aiemjoy et al., 2022), antibody secreting cell models (White et al., 2019, Pelleau et al., 2021, White et al., 2014, White et al., 2015, Yman et al., 2019), and phenomenological models (Zhao et al., 2018, Teunis et al., 2002). Theoretical work has explored the relevance of different timescales of short and long-term antibody secreting cells in explaining serological patterns (Antia et al., 2018, Amanna and Slifka, 2010, Amanna et al., 2007), though in practice biological realism and complexity must be balanced against parameter identifiability and avoiding overfitting (Andraud et al., 2012, Fraser et al., 2007).

The within-host model need not be limited to a single biomarker, and extensions to describe multiple biomarker kinetics simultaneously can improve TSI estimate precision (Borremans et al., 2016, Rydevik et al., 2016, Prager et al., 2020, Giardina et al., 2019). This might involve: modeling antibody kinetics against multiple antigens (Pelleau et al., 2021, Garcia et al., 2022, Yman et al., 2019), which may provide orthogonal information on TSI; combining measurements of multiple antibody classes which undergo boosting and waning at different times post infection (Simonsen et al., 2009, Simonsen et al., 2008); or by considering antibody avidity in addition to titer, which often continues to increase over time (Garcia et al., 2022, Gray et al., 1993).

A large amount of variation in biomarker kinetics cannot be explained by TSI alone. Differences across pathogens, exposure types, host factors, individual-level variation, infection history and the biochemistry of target biomarkers all require consideration (Table 5). For example, for malaria, a eukaryotic organism that encodes >5000 genes, discovery of biomarkers associated with recent exposure is an ongoing area of research (Greenhouse et al., 2018). It is important to consider if differences observed between groups in serological datasets (e.g., by age) are a reflection of heterogeneities in within-host kinetics, or if they reflect genuine differences in exposure history between groups (Mosterín Höpping et al., 2016). Choosing which sources of heterogeneity to account for when designing a within-host model will depend on the research question, availability of data to power a complex model, and an understanding of which sources of variation are most impactful on the observed serological data.

#### 4.2.2 The epidemiological level: models and priors for the unknown time-since-infection

Many approaches, particularly those early TSI methods developed for HIV, take a two-stage approach to estimating the force of infection or incidence rate using individual TSI estimates. Each individual’s TSI is estimated independently, and subsequently combined to draw inferences at the population-level (Pepin et al., 2017, Murphy and Parry, 2008, Prager et al., 2020). This approach makes no explicit assumptions about the infection-generating process while estimating TSI and is suitable in systems where infection risks are uncorrelated (e.g., individuals come from separate populations), or where the biomarker data is sufficient to provide very precise TSI estimates.

Although it is possible to consider the infection-generating process as part of this two-step process (Wilber et al., 2020, Pepin et al., 2019), representing the force of infection explicitly as part of the full TSI model can be helpful and sometimes necessary to avoid bias when reconstructing incidence curves (Menezes et al., 2023) (Appendix 2.2). Simple serocatalytic and TSI models often assume that the FOI is constant (e.g., infections arise under a Poisson process) (Borremans et al., 2016, Simonsen et al., 2009, Rydevik et al., 2016, Aiemjoy et al., 2022). However, this assumption clearly does not hold for pathogens which exhibit strong epidemic dynamics or seasonality (Salje et al., 2018, Tsang et al., 2022). Explicitly incorporating more complex epidemiological models into the TSI framework has a number of benefits including: a) shrinkage of infection time estimates by borrowing information from other individuals; b) incorporation of individual TSI uncertainty and prior information into force of infection estimates; c) direct estimation of event risk (e.g., immunity, disease risk) as a function of time-since-infection or latent biomarker levels (Salje et al., 2021, Salje et al., 2018, Ranjeva et al., 2018). This is particularly important when the binary infection state is unknown and to be imputed for each individual, where the model structure can place strong prior information on if and when an individual was infected (Kucharski et al., 2018, Hay et al., 2020).

### 4.3 Time-since-infection methods with multiple exposures and variants

There are many pathogen systems where serological data reflect the culmination of multiple infections and vaccinations, either due to waning immunity, a non-sterilizing primary immune response, or antigenic variation leading to immune escape. Influenza and coronaviruses are classic examples of antigenically variable pathogens (Smith et al., 2004, Mykytyn et al., 2023, Kucharski et al., 2015a), though many others such as the flaviviruses (Priyamvada et al., 2016, Katzelnick et al., 2015, Katzelnick et al., 2021), norovirus (Lindesmith et al., 2022), *Streptococcus pneumoniae (*Auranen et al., 2000; Geno et al., 2015), and malaria (Gupta and Day, 1994, Recker et al., 2004) also exhibit antigenic variation and reinfection. Inference of infection timings, within-host kinetics and population-level epidemiology using serological data is still possible using the same basic time-since-infection framework, but with modifications to the within-host and population-level models (Fig. 2D). There are currently few frameworks for estimating multi-exposure epidemiological dynamics using multi-antigen serology panels, though the inference objective is the same as the broad time-since-infection approach described above.

#### 4.3.1 The within-host level with multiple exposures and variants

The kinetics of the immune response may differ between re-infection and primary infection, both in terms of speed and the relative contributions of recall versus *de novo* responses. Depending on how well recognized the infecting pathogen is by the host’s immune history, this mixture of recall and novel responses can lead to complex immunological phenomena such as different kinetics between primary and subsequent exposure, original antigenic sin (Cobey and Hensley, 2017, Lessler et al., 2012), back-boosting (Fonville et al., 2014), immune imprinting (Oidtman et al., 2021), antibody-dependent enhancement (Whitehead et al., 2007, Halstead et al., 1983), immunodominance (Angeletti et al., 2017) and epitope masking (Zarnitsyna et al., 2015). In practice, these mechanisms often do not need to be modeled explicitly and can be simplified by assuming that the antibody response following secondary exposure is conditional only on the pre-infection antibody level (e.g., accounting for antibody ceiling effects (Ranjeva et al., 2018)). It is also common to ignore the difference between primary and reinfection kinetics when data are insufficient to justify additional model complexity (Salje et al., 2018, White et al., 2014).

As with binary serostatus data, for antigenically variable pathogens cross-reactivity is also a challenge for interpreting quantitative serological data. For example, elevated antibody titers against Mayaro virus (MAYV) might reflect Chikungunya virus (CHIKV) and/or Mayaro virus (MAYV) infection in areas where the two alphaviruses co-circulate, and thus the presence of CHIKV infection would need to be ruled out or estimated jointly with the MAYV infection (Hozé et al., 2020). In the case of influenza, Kucharski et al. demonstrated how external data on antigenic relatedness could be included in an inference model to disentangle the contributions of multiple lifetime infections to observed multi-strain antibody profiles, allowing the estimation of individual-level lifetime infection histories (Kucharski et al., 2018, Hay et al., 2020, Hay et al., 2024).

#### 4.3.2 The epidemiological level with multiple exposures and variants

The number, timing, and identity of potential exposure histories and the number of parameters to estimate grows rapidly with the number of possible reinfections for multi-variant pathogens (Kryazhimskiy et al., 2007, Kucharski et al., 2015b). There are currently three approaches taken in the literature to estimating multiple exposures: a) exposure timings are fixed either based on longitudinal serological data or confirmed infection using syndromic or laboratory diagnostics (Kucharski et al., 2018, Hay et al., 2020); b) the number of potential infections is set in advance, but their timing and identity are estimated, such that the number of parameters to be estimated is fixed prior to attempting any inference (Wilber et al., 2020); c) reversible-jump Markov chain Monte Carlo (RJMCMC) is used to jointly estimate both the number of model parameters and their values (Salje et al., 2018, Tsang et al., 2022, Hodgson et al., 2024). Although straightforward in principle, these approaches can be challenging to implement in practice, and there are currently very few inference frameworks which make use of industry-standard tools such as Stan  (Antia et al., 2018, Amanna et al., 2007).

Despite these challenges, incredibly detailed insights can be drawn about individual-level and population-level epidemiology of antigenically variable pathogens using serological data. One key example comes from Salje et al., who developed a RJMCMC framework to impute unobserved dengue infections from changes in antibody titers, estimating both their timing and likely serotype (Antia et al., 2018, Amanna et al., 2007, Kucharski et al., 2015b). This study used a rich dataset of longitudinal antibody titer measurements against multiple dengue serotypes from over 3000 individuals, many of whom had PCR-confirmed infections which could anchor post-infection antibody kinetics for a subset of infections. This mixture of time-varying antibody titers with and without known TSI provided information to back-calculate the timing of unobserved infections from changes in antibody titer. The richness of the dataset allowed for a complex multilevel model to be fitted, accounting for many of the sources of variation listed in Table 5 including individual-level heterogeneity in response, assay variability, a time-varying, serotype-specific force of infection, and infection and disease risk conditional on current antibody titer. Although it is rare to have a dataset sufficient to estimate so many parameters, this study demonstrates the level of insight that can be drawn from multi-strain serological data using complex TSI methods.

## 5 Future directions and upcoming developments in serodynamics

With the increasing availability of high throughput, multiplex serological assays, future methods development in serodynamics cannot rely solely on classic mathematical and statistical modeling. In this section, we first give an overview of key use cases for machine learning methods where epidemiological or immunological questions can be answered using predictive models rather than requiring an understanding of underlying mechanism. Finally, we discuss the need to incorporate immunity, rather than just serology, into serodynamics methods, and the importance of harmonizing data reporting and serodynamics toolkits.

### 5.1 Addressing complex immunological and epidemiological questions using machine learning methods

The aim of the methods described so far is to estimate key epidemiological parameters using mathematical models capturing the data-generating process. However, many pathogen and immunological systems are extremely difficult to model explicitly. For example, many pathogens lack validated biomarkers for exposure recency, and thus which biomarkers to measure to backcalculate exposure timing may be unknown. In these cases, supervised machine learning approaches are useful for identifying minimal sets of biomarkers to predict or classify past infections and vaccinations (Pelleau et al., 2021, Arnold et al., 2017, Jones et al., 2022, Rosado et al., 2021b, Wiens et al., 2023, Radtke et al., 2021, Oyamada et al., 2021) (Fig. 2E). This is particularly important for pathogens where cross-reactivity with off-target pathogens is likely, or where substantial variation in serological signatures are expected, making serocatalytic models and time-since-infection methods very difficult to parameterize (Neto da et al., 2022, Thao et al., 2020, Sangkaew et al., 2020). Machine learning methods are also useful for analyzing protein microarray or other high-dimensional serological data from immunologically complex pathogens such as malaria, where the large number of antigens and the expression of different antigens depending on the life-stage of the parasite makes modeling the kinetics of antibodies targeting each antigen prohibitively complex (Helb et al., 2015, Corran et al., 2007, Arnold et al., 2014, Longley et al., 2020).

A key direction for further methods development is to use individual-level TSI estimates from machine learning methods to estimate population-level infection trends, particularly where clinical surveillance is lacking (Arnold et al., 2017, Fountain-Jones et al., 2019). For example, multiplex assays such as PepSeq can be used to identify individual infection histories (i.e., using longitudinal sera) to multiple pathogens tested on a single assay, but extrapolating these individual events to the population-level requires further modeling (Kelley et al., 2023). Azman et al. developed one such framework to combine machine learning-based TSI estimates with epidemiological simulations to reconstruct epidemic curves for cholera (Azman et al., 2019). Azman et al. trained a random forest model on over 1500 confirmed cholera cases to categorize recency of infection from a panel of IgG, IgM and IgA measurements against various antigens and vibriocidal assays, and then used simulations to demonstrate how these machine learning-derived infection states could be used to back-calculate cholera incidence using cross-sectional serology. Combining bioinformatics pipelines for multiplex data using machine learning algorithms with epidemiological modeling to estimate incidence trends and the force of infection is a key direction for further research in serodynamics.

Machine learning approaches for estimating infection timing do have limitations. First, unlike traditional within-host kinetics models, it is difficult to interpret the fitted model in terms of biologically interpretable parameters, complicating the ability to make longer-term predictions based on extrapolating model predictions. This also means that within-host kinetics are not easily constrained *a priori* based on the model structure. Second, the dependence on training data from a particular cohort or assay makes overfitting and poor generalizability a risk compared to simpler models fitted to data from standardized assays.

### 5.2 Linking serodynamics to population immunity

While this review has focused on methods for estimating epidemiological trends and estimating past infections and vaccinations, a key direction following naturally from modeling antibody dynamics is to link antibody landscapes to population immunity (Yuan et al., 2017, Kucharski and Baguelin, 2017). For pathogens with well-defined correlates or surrogates of protection, which are readily measured using routine serological assays, transforming antibody levels into susceptibility measures can be as straightforward as transforming titers into relative risks of infection (e.g., protection landscapes in (Plotkin, 2010); Kucharski and Baguelin, 2017) (Plotkin, 2010, Plotkin, 2022). However, this is challenging in practice, as reliable correlates against infection do not exist for all pathogens (Satti et al., 2022, Langhorne et al., 2008, Tran et al., 2013) and correlates may differ between individuals conditional on covariates such as exposure history, variant or age (Wei et al., 2022, Black et al., 2011, Khoury et al., 2023). One promising approach when there is not a single well-defined correlate of protection is to use multivariate models to evaluate multiple correlates of protection and estimate their independent and synergistic contributions to immune protection (Mettelman et al., 2023). Linking serology to immunity is therefore an ongoing area of research alongside improved measures and understanding of relevant immune markers (e.g., the relative contributions of IgA and IgG to protection (Hertz et al., 2023); Liew et al., 2023). Progress in this area will be key to incorporating serology into epidemiological prediction tasks such as epidemic forecasting and outbreak risk assessment (Cheung et al., 2022, Johansson et al., 2019).

### 5.3 Harmonizing serological data collection and analysis

There have been many promising advances in the analytics and technologies for generating detailed insights into epidemiological processes from serological data. The standardization of methods and compilation of comparable serological datasets has been proposed recently through a “Global Immunological Observatory” (GIO): a system to collect, store and process serological samples at a broad scale to quickly detect new outbreaks and to characterize global population immunity (Metcalf et al., 2016, Mina et al., 2020). Such a system may become feasible with the rise of data sharing through online dashboards, sample collection and the ability to process sera at scale (Azman et al., 2019, Mina et al., 2020). The broader field of seroepidemiology could look to key examples from influenza serology, where there have been long-standing efforts to standardize laboratory assays and protocols (Mina et al., 2020, Pearson and Lipman, 1988), and from genomics and bioinformatics, where there have been efforts to harmonize data standards and create interoperable analytical software (Pearson and Lipman, 1988, Gentleman et al., 2004).

Going forward, there are two key considerations for creating standardized serodynamics toolkits. First, the increasing availability of multiplex and epitope-level serological assays presents challenges in relating panels of complex antibody profiles to individual histories of exposure and future susceptibility. The technologies to generate complex antibody profiles to thousands of epitopes from multiple pathogens at once, such as the phage immunoprecipitation array VirScan, have been used to understand immune amnesia following measles infection (Mina et al., 2019), identify pan-coronavirus-reactive antibodies (Shrock et al., 2020), and identify HIV-1 and hepatitis C virus infection simultaneously from a single assay run (Hada-Neeman et al., 2021). These data are more difficult to directly link to functional immunity, as they are typically based on capturing serum antibodies that bind to individual phage expressing single epitopes rather than measures of antibody concentration or function. Machine learning approaches are likely to be particularly useful for analyzing these data, though mechanistic models linking sequencing read counts to within-host processes will also be useful. An additional area not discussed in this review is measures beyond serum antibodies, including systems serology (Chung et al., 2015) approaches measuring antibodies of different classes and functions, profiling T-cell and B-cell repertoires at scale (Kula et al., 2019, Deering et al., 2023, Quadeer et al., 2021), or high-throughput systems immunology approaches measuring innate, cellular and humoral compartments (Hu et al., 2022, Forlin et al., 2023). Modeling work applying these multi-modal types of data to answer epidemiological questions is still extremely limited.

Second, there is a need to standardize and make more accessible serodynamics tools and inference methods. Decisions made in the upstream bioinformatic methods used to pre-process laboratory data affect the downstream epidemiological inference, and guidelines and best practices are needed. Mixture models and serocatalytic models for analyzing binary serostatus data are methods that have been widely used in research settings, with a number of open source statistical packages now available to implement them (Hens et al., 2012, Estimates, 2023, Hoze, 2023). Time-since-infection methods are also now becoming more widely available (Hoze, 2023, Yuan et al., 2017), but more complex methods frameworks are still largely implemented as boutique codebases for particular datasets. Many modeling approaches rely on custom samplers or optimization algorithms, which limits their widespread use.

Finally, serodynamics remains a relatively specialist field, where methods are primarily custom-built and used by academic researchers, which limits potential adoption and use by public health decision-makers and other stakeholders. Standardized approaches and clear training materials may expand the accessibility and appropriate use of serological data, allowing a variety of epidemiological questions to be answered for different pathogens and contexts. A clearer understanding of the types of questions that can and should be answered with serology could lead to new opportunities for serological data collection and the incorporation of results into public health decision-making.

## 6 Conclusions

While both binary and quantitative serological data can be extremely useful for informing public health decision making and epidemiological surveillance, the full utility of serological data cannot be realized without appropriate approaches for extracting epidemiological insights. In this review, we have provided an overview of key approaches to analyze serological data, depending on the data types available and the epidemiological questions of interest. In this complex landscape, there is value in understanding the equivalencies and differences between modeling approaches and the assumptions they make, as well as moving towards standardized frameworks for analysis of serological data. This can enable better comparison between analyses and datasets, due to the use of comparable model parameters, and by adjusting analyses to account for different assays and populations between studies. This in turn enables stronger and more robust evidence synthesis for epidemiological insights.

## CRediT authorship contribution statement

**James Hay:** Writing – review & editing, Writing – original draft, Visualization, Validation, Supervision, Software, Resources, Project administration, Methodology, Investigation, Funding acquisition, Conceptualization. **Saki Takahashi:** Writing – review & editing, Writing – original draft, Visualization, Validation, Supervision, Software, Resources, Project administration, Methodology, Investigation, Funding acquisition, Conceptualization. **Isobel Routledge:** Writing – review & editing, Writing – original draft, Visualization, Validation, Supervision, Software, Resources, Project administration, Methodology, Investigation, Conceptualization.

## Declaration of Competing Interest

The authors declare the following financial interests/personal relationships which may be considered as potential competing interests: James Hay reports financial support was provided by Wellcome Trust. Saki Takahashi reports financial support was provided by Bill & Melinda Gates Foundation. If there are other authors, they declare that they have no known competing financial interests or personal relationships that could have appeared to influence the work reported in this paper.

## Data Availability

No data was used for the research described in the article.
