# Supplementary material for: Serodynamics: A primer and synthetic review of methods for epidemiological inference using serological data
Source: Epidemics. 2024 Dec;49:100806. doi: 10.1016/j.epidem.2024.100806 (PMC11649536; doi:10.1016/j.epidem.2024.100806)
Supplement: Figure S1 — Supplementary material. [file mmc1.docx]

**Appendix for “Serodynamics: A primer and synthetic review of methods for epidemiological inference using serological data”**

*Linking seroepidemiological models by considering the data-generating process*

Much of infectious disease modeling is derived from, or at least shares, key concepts with survival analysis. The hazard function, describing the failure rate (or infection rate in the context of infectious disease models), and the survival function, describing the probability of survival (or avoidance of infection) beyond a given time can be used to link all the seroepidemiological models described in the main text. Further links with survival analysis and more complex force of infection models can be derived and are discussed in [1,2] and independently in [3]. For the purposes of this review, we demonstrate the link between serocatalytic and time-since-infection models through the convolution of prevalence, incidence, and within-host processes.

There are two key concepts which provide intuition behind most methods for inferring epidemic dynamics from serological data: within-host kinetics and the data-generating process. First, within-host kinetics refers to how individual biomarker levels, usually antibody titers or concentrations (but also antibody avidities, lymphocyte counts, viral loads, parasite densities, etc.), change somewhat predictably over time following infection and vaccination (**Figure S1**). A serum sample therefore reflects only a snapshot in time of an individual’s continually changing immune profile. Understanding how measured biomarker levels change over time – and the implications for interpreting serological assay outputs – is therefore essential when modeling serological data. It is also important to note that biomarker kinetics and assay targets vary across pathogens, and thus adapting seroepidemiological models to new pathogen systems must begin with immunological understanding.

Second, the data-generating process refers to the set of immunological and ecological processes which occur at different scales and interact to generate observed serological data (summarized in **Figure S2** and [4]). The timescales and study designs that feed into different analytical approaches can vary; however, all of these approaches either explicitly or implicitly assume some model for the multi-level process which determines the distribution of observed serological data. These levels are: (1) an epidemiological level, where exposures to a pathogen are hidden events occurring at a rate governed by the force of infection and dampened by host immunity; (2) the within-host level, where exposures lead to the stimulation of an immunological response and subsequent biomarker kinetics; (3) the observation level, where serological surveys sample and observe snapshots of this within-host process through antibody or biomarker measurements which are inherently noisy. Much of the challenge in designing and implementing a serodynamics framework is in identifying and modeling the key mechanisms of the data-generating process which relate observed serological data to model parameters and statistics. Framing various serodynamics methods within a common data-generating process makes clear their similarities, despite their different presentation in the literature. For example, the simple serocatalytic model can be thought of as a simplified scenario of a more general model describing the distribution of quantitative antibody levels conditional on some incidence curve and within-host kinetics model.


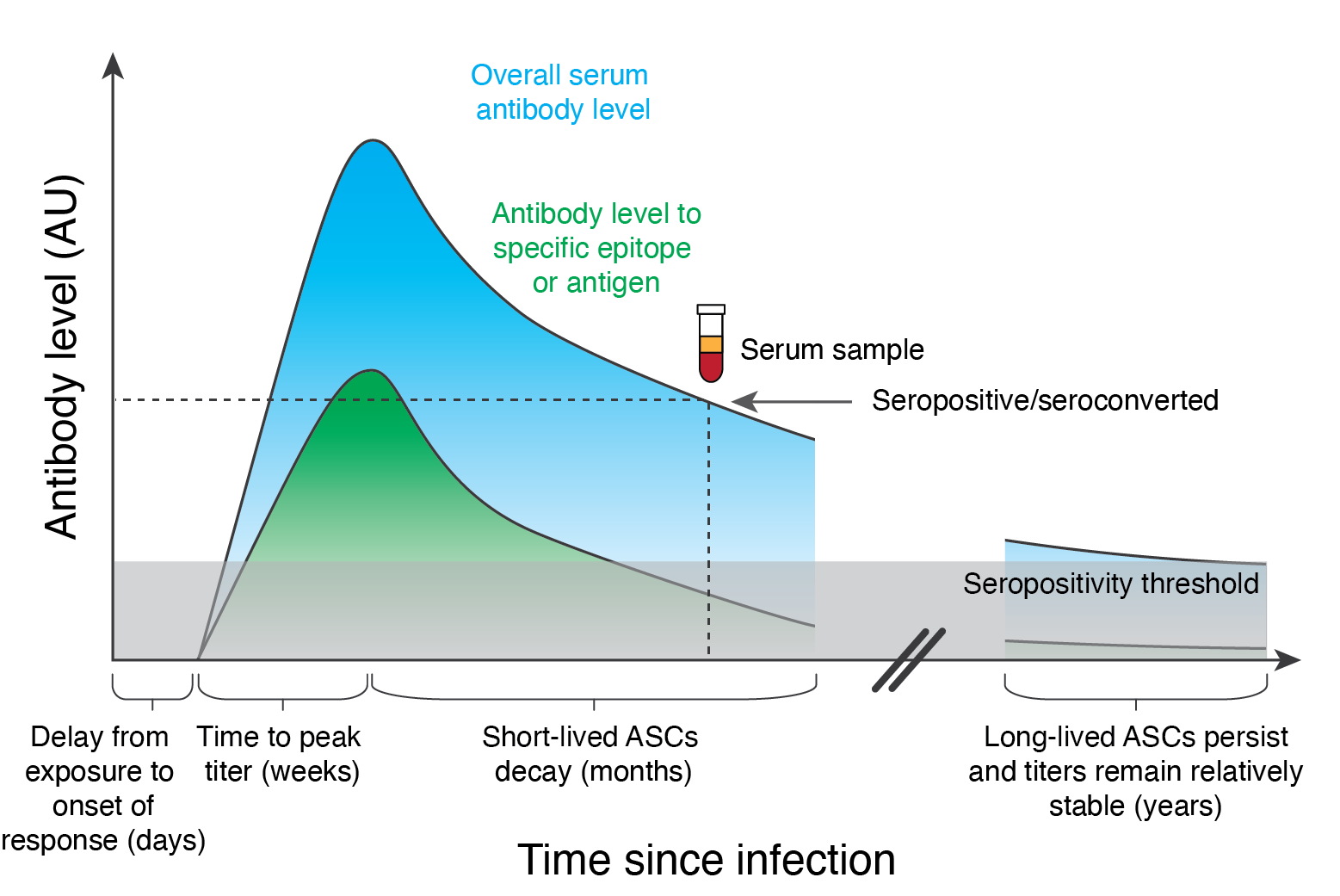


**Figure S1:** **Antibody kinetics following a single exposure event.** Serum antibody levels increase following infection as the host’s immune system mounts a humoral response. Antibody levels take time to increase and peak and subsequently wane over time in short- and long-term phases [5]. Observed kinetics depend on the type of assay used and the target biomarker. For example, overall serum antibody levels (e.g., a neutralization assay) reflect the presence of a polyclonal antibody response to many antigens, whereas an epitope- or antigen-specific assay will reflect antibody levels to a subset of the overall antibody response against a pathogen. The horizontal, dashed line illustrates how an antibody assay measurement may lead to a sample being identified as seropositive in the ideal case. However, if a sample is taken too late or early in the response, or the kinetics of the target biomarker have not passed the seropositivity threshold, then the individual may be incorrectly classified as immunologically naive.

**
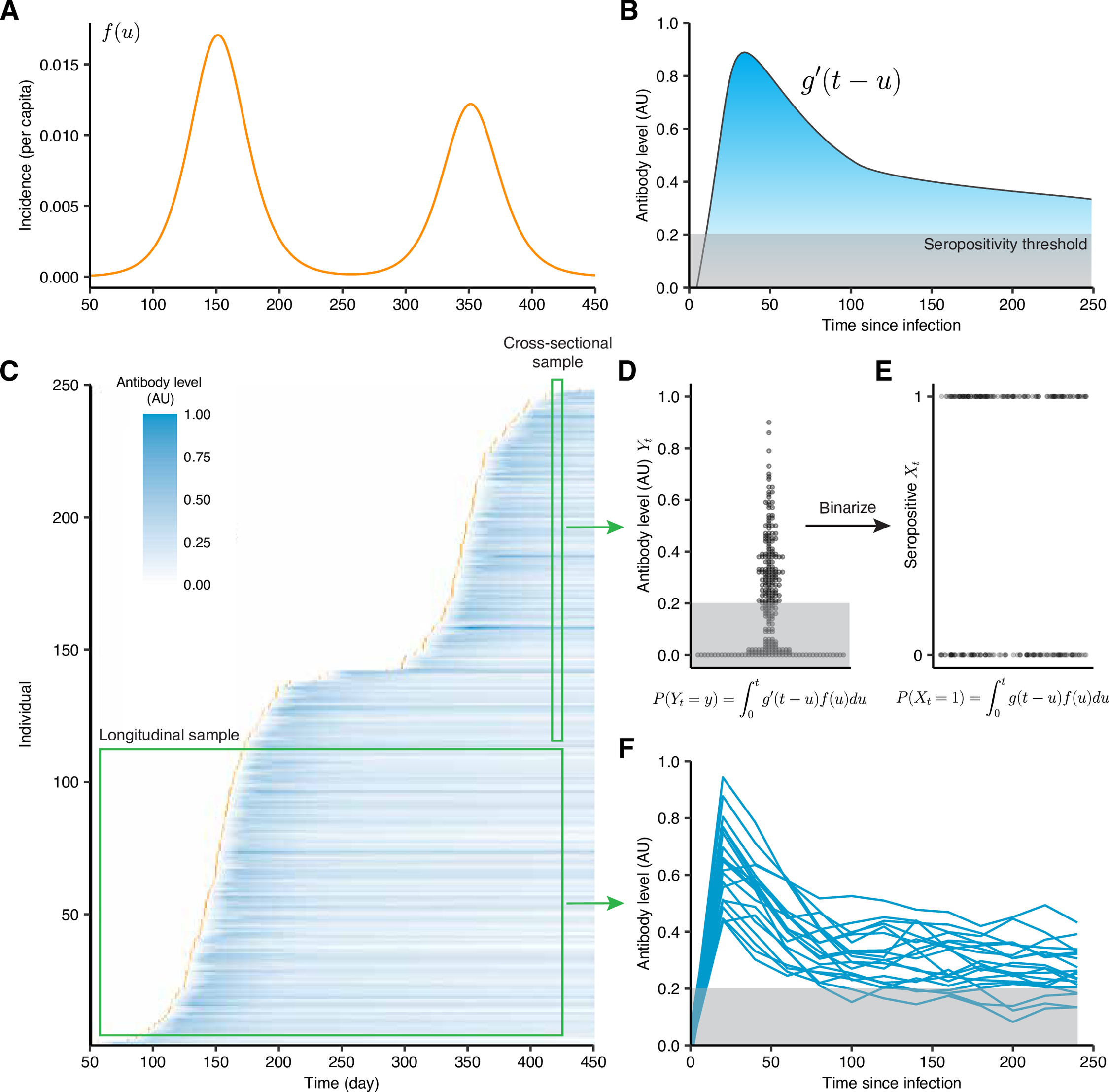
**

**Figure S2: The data-generating process for serological data.** Serological data reflect the convolution of within-host and population-level processes. (A) The incidence of infection over time *u*, *f(u),* determines the distribution of times-since-infection, here exemplified as a two-peak epidemic. (B) Antibody levels follow predictable boosting and waning kinetics following infection as described in **Figure S1**. The probability of observing an antibody level *Y_t_* at time *t* is therefore conditional on the time-since-infection, *g’(Y_t_|t-u)*. (C) The distribution of individual antibody levels in the population is determined by the infection generating process (A), which determines the timing of infections (orange dots), combined with the within-host kinetics process, which determines the expected antibody level for an individual over time (blue streaks). Different sampling strategies capture different slices of this underlying antibody level distribution. (D) A cross-sectional sample gives a distribution of antibody titer measurements reflecting the convolution of the incidence curve, *f(u),* and the within-host kinetics model, *g’(Y_t_|t-u)*, which is related to seroprevalence (E) simply by replacing the antibody kinetics model with a seropositivity model, *g(t-u)*. (F) Longitudinal studies with well-characterized infection times measure individual-level antibody trajectories over time-since-infection, which may be highly varied.

**Definitions**

- $Z_{t}=\{0,1\}$ infection state, 1 if *ever* infected by time *t*, 0 if never infected by time *t*
- $X_{t}=\{0,1\}$ current positivity or infection state (i.e., detectable on a serologic or diagnostic test), 1 for positive, 0 for negative
- $Y_{t}=$ observed biomarker level at time *t*
- $t_{i}=$ infection time
- $t-u=$ time-since-infection given observation at time *t* and infection at time *u*

**1. The link between incidence, prevalence and the serocatalytic model**

**1.1 Incidence**

Let the hazard of infection on a given day (i.e., per-capita incidence per unit time) be given by $f(t)$ (**Figure S2A)**. Although $f(t)$ is a hazard by definition, we can treat it here as a probability density function by excluding reinfections. The cumulative incidence, $I\left( t \right)$, is therefore given by:

Equation 1

$$P\left( Z_{t}=1 \right)=I\left( t \right)= \int_{0}^{t} f\left( u \right)du\text{ }$$

The probability of having been infected at time *u,* given observation at time *t* and given the individual has been infected, $Z_{t}=1$, is:

Equation 2

$$P\left( t_{i}=u|Z_{t}=1 \right)=\frac{f(u)}{\int_{0}^{t} f\left( u \right)du\text{ }}$$

Note $P\left( t_{i}=u|Z_{t}=0 \right)=0$, assuming that $u<t$. We can then marginalize over the infection state parameter $Z_{t}$ to recover the incidence function. Although trivial, showing this explicitly will be useful later:

Equation 3

$$P\left( t_{i}=u \right)=P\left( t_{i}=u|Z_{t}=1 \right)P\left( Z_{t}=1 \right)+P\left( t_{i}=u|Z_{t}=0 \right)P\left( Z_{t}=0 \right)$$

= $\frac{f(u)}{\int_{0}^{t} f\left( u \right)du\text{ }}$ $\int_{0}^{t} f\left( u \right)du\text{ }$

$$=f(u)$$

- 1. **Prevalence**

Let $X_{t}$ denote an individual’s current infection state, or current test positivity state (1 if currently infected/currently testing positive, 0 otherwise), and *u* represent the timing of infection:

Equation 4

$$P\left( X_{t}=1 | Z_{t}=1,t_{i}=u \right)=g(t-u)$$

Where $g(t-u)$ is the probability of testing positive given time-since-infection *t-u* days i.e., the probability of having not seroreverted. Note that $P(Z_{t}=1$| $t_{i}=u)=1$, $P(Z_{t}=$0| $t_{i}=u)=0$ and $P\left( X_{t}=1 | Z_{t}=0 \right)=1-sp$ accounting for imperfect test specificity, $sp$. Simple models assume that all individuals remain positive for the biomarker being measured for a fixed duration (sometimes called the *window* period e.g., [6]), whereas more complex models can account for complex within-host kinetics and imperfect sensitivity and specificity, which may vary by exposure type, biomarker and individual.

Because the timing of infection is almost always unobserved, the time-since-infection parameter is usually integrated out:

Equation 5

$$P\left( X_{t}=1 | Z_{t}=1 \right)=\frac{\int_{0}^{t} g\left( t-u \right)f\left( u \right)du}{\int_{0}^{t} f\left( u \right)du}$$

Finally, marginalizing out the infection state parameter, $Z_{t}$, we get the observed prevalence, $\pi(t)$ (**Figure S2E** if $sp=1$):

Equation 6

$$\pi\left( t \right)=P\left( X_{t}=1 \right)=P\left( X_{t}=1 | Z_{t}=1 \right)P\left( Z_{t}=1 \right)+P\left( X_{t}=1 | Z_{t}=0 \right)P(Z_{t}=0)$$

$$=\int_{0}^{t} g\left( t-u \right)f\left( u \right)du+(1-sp)(1-\int_{0}^{t} f\left( u \right)du)$$

The intuition is that the number of individuals who are currently seropositive (or infected if using a more transient biomarker for infection state) today is given by the culmination of individuals who were infected over all past times, multiplied by the proportion of those individuals who remained (sero)positive up until today. Cumulative incidence is a special case if infection or test positivity is assumed to be lifelong (i.e., $g\left( t \right)=1$ for all *t*).

**1.3 Link to the serocatalytic model**

If we assume that infections arise under a constant force of infection, $\lambda$ (equivalent to the hazard rate in survival analysis), then the probability of remaining susceptible by time $t$, $S\left( t \right)$, is then given by:

Equation 7

$$S\left( t \right)=e^{-\lambda t}$$

The instantaneous probability of infection at time *t* is then given by:

Equation 8

$f\left( t \right)=-\frac{dS\left( t \right)}{dt}=\lambda S\left( t \right)=$ ${\lambda e}^{-\lambda t}$

Substituting this into the cumulative incidence equation, $P(Z_{t}=1)$, we recover the familiar serocatalytic model/survival function:

Equation 9

$$\pi\left( t \right)=P\left( Z_{t}=1 \right)=\int_{0}^{t} f\left( u \right)du=\int_{0}^{t} {\lambda e}^{-\lambda u}du=1-e^{-\lambda t}$$

The interpretation is straightforward – the probability of an individual becoming seropositive by time $t$ is the complement of the probability that the individual avoided infection up until that time. Note that time ($t$) can be replaced with age ($a$) in the above equation by measuring changes in seroprevalence in a cohort across age groups rather than over time, as the model uses information on the cumulative time the population was at risk of seroconversion (**Figure S3**). Age is more commonly used than time to inform on dynamics over time, as it is challenging to carry out multiple cross-sectional serological surveys over long periods of time.

**
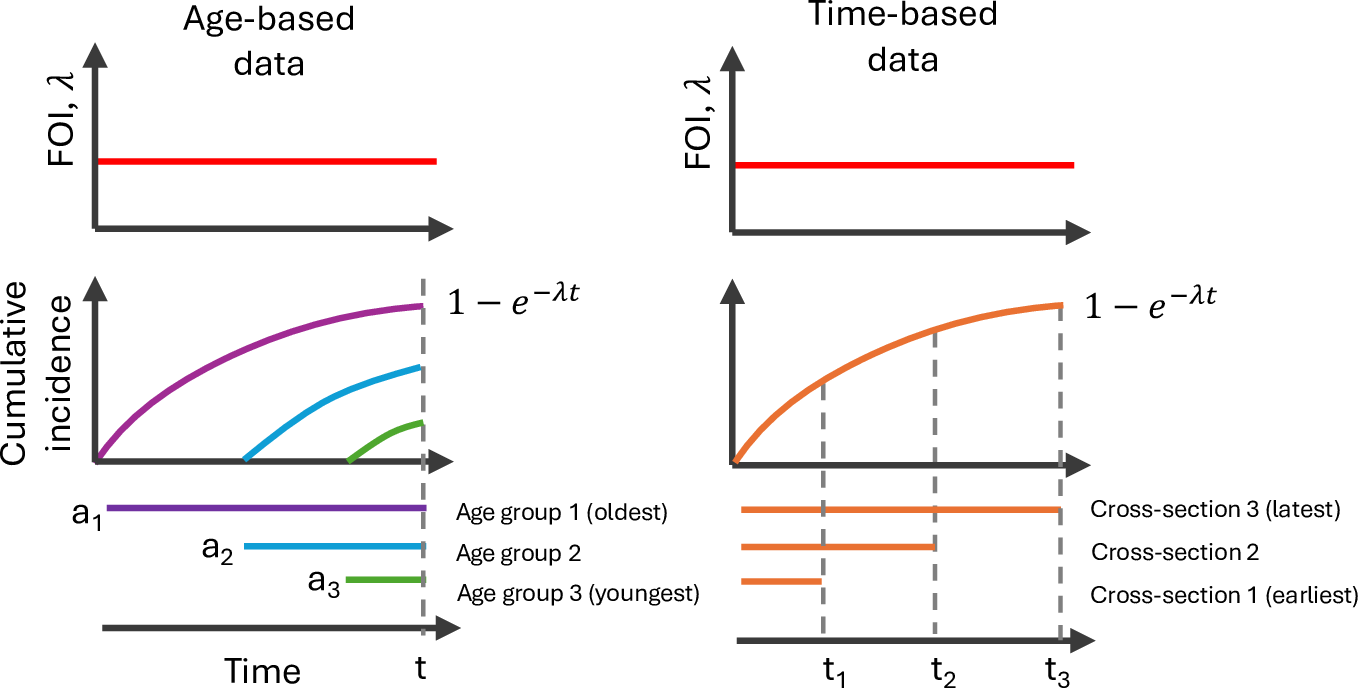
**

**Figure S3: Comparison of serocatalytic models fitted to age-stratified or time-stratified cross-sectional data.** With age-based data, the FOI is informed by differences in seroprevalence (cumulative incidence) between age groups, which reflects differences in the time spent at risk of infection. With time-based data, serial cross-sections capture individuals of mixed ages, where later cross-sections reflect individuals who have been at risk of infection for longer than those from earlier cross-sections.

Thus, there is a direct link between survival analysis, the convolutional relationship between prevalence and incidence, and the simple serocatalytic model. Of course, more complex assumptions regarding the force of infection [7], the duration of positivity etc. can be assumed. Note that care must be taken to account for the duration that each individual is at risk, which may not match the time period of interest or may be different between individuals (censoring by birth date, death, etc.) [3].

The assumption of a constant force of infection can be relaxed and the model re-written as:

Equation 10

$$\pi\left( t \right)=1-e^{-\int_{0}^{t} \lambda(u)du}$$

Or where the sample contains individuals of different ages:

Equation 11

$$\pi\left( a,t \right)=1-e^{-\int_{t-a}^{t} \lambda(u)du}$$

Whereas the FOI parameter gives the rate of seroconversion, an additional parameter, usually denoted $\rho$, can also be included to model the rate of the reverse process, seroreversion (where $1/\rho$ gives the mean duration of seropositivity). The reversible serocatalytic model can then be expressed as:

Equation 12

$$\pi\left( a \right)=\frac{\lambda}{\lambda+\rho}(1-e^{-a\left( \lambda+\rho\right)})$$

**2. Population-level serodynamics**

**2.1 The population-level distribution of biomarker levels over time**

It is straightforward to extend the model above to consider not only the probability of being infected or positive at time $t$, but instead the probability of observing an antibody titer $Y_{t}$ at time $t$ simply by replacing the binary infection or serostatus $X_{t}$ with the antibody level $Y_{t}$, and replacing $g(t)$ with a function describing the distribution of (detectable) antibody levels $Y_{t}$ at time $t$ after infection at time $u$ (**Figure S2B**). This within-host model can be generically described by the distribution of biomarker levels at time $t$, $Y_{t}$, conditional on the individual’s time-since-infection:

Equation 13

$$P\left( Y_{t}=y | t_{i}=u,Z_{t}=1 \right)=g'(t-u)$$

Where $t-u$ denotes the time between an exposure event and the observation time (i.e., the time-since-infection), $g'(t-u)$ gives the distribution of biomarker levels given the time-since-infection for previously infected individuals (closely related to the probability of remaining infected/positive, as we will see below).

As above, the infection state parameter $Z_{t}$ can be marginalised out easily, given that the probability of not being infected given the time-since-infection is zero:

Equation 14

$$P\left( Y_{t}=y | t_{i}=u \right)=P\left( Y_{t}=y | t_{i}=u,Z_{t}=1 \right)P\left( Z_{t}=1 | t_{i}=u \right)+ \left( Y_{=yt} | t_{i}=u,Z_{t}=0 \right)P\left( Z_{t}=0 | t_{i}=u \right)$$

$$=g'(t-u)$$

If we instead marginalise over the time-since-infection parameter but condition on being infected:

Equation 15

$$P\left( Y_{t}=y | Z_{t}=1 \right)=\int_{0}^{t} P\left( Y_{t}=y | t_{i}=u,Z_{t}=1 \right)P\left( t_{i}=u | Z_{t}=1 \right)du$$

$$=\frac{\int_{0}^{t} g^{'}\left( t-u \right)f\left( u \right)du}{\int_{0}^{t} f\left( u \right)du}$$

It is then easy to see how the time-varying distribution of biomarkers are linked to prevalence if we also marginalise over the infection state parameter (**Figure S2D**):

Equation 16

$$P\left( Y_{t}=y \right)=P\left( Y_{t}=y | Z_{t}=1 \right)P\left( Z_{t}=1 \right)+P\left( Y_{t}=y | Z_{t}=0 \right)P(Z_{t}=0)$$

$= \frac{\int_{0}^{t} g^{'}\left( t-u \right)f\left( u \right)du}{\int_{0}^{t} f\left( v \right)dv}\int_{0}^{t} f\left( u \right)du+h()(1-\int_{0}^{t} f\left( u \right)du$*)*

$=\int_{0}^{t} g^{'}\left( t-u \right)f\left( u \right)du+h()(1-\int_{0}^{t} f\left( u \right)du$*)*

where $h()$ gives the distribution of biomarker levels for naïve individuals, which does not depend on the time-since-infection and accounts for imperfect specificity when measuring *Y*.

It is important to note that the distribution of biomarker levels in a single cross-section is a mixture distribution of observations from infected and not infected individuals, which highlights the link between more complex inference methods and approaches using mixture models [8]. However, this formulation also highlights that the distribution of observations from infected individuals is itself a mixture distribution with contributing components from each possible time-since-infection, weighted by the incidence at each time ($f\left( u \right))$ with expectations depending on the time-since-infection ($g^{'}\left( t-u \right)$) (**Figure S4**). This mixture distribution for infected individuals does not have a closed-form solution except under very simple assumptions for these two models (e.g., constant FOI and biomarker levels independent of time-since-infection), and thus in practice it is usually necessary to model discrete rather than continuous time and to numerically sum over the contribution of infections between time *0* and *t.*

**
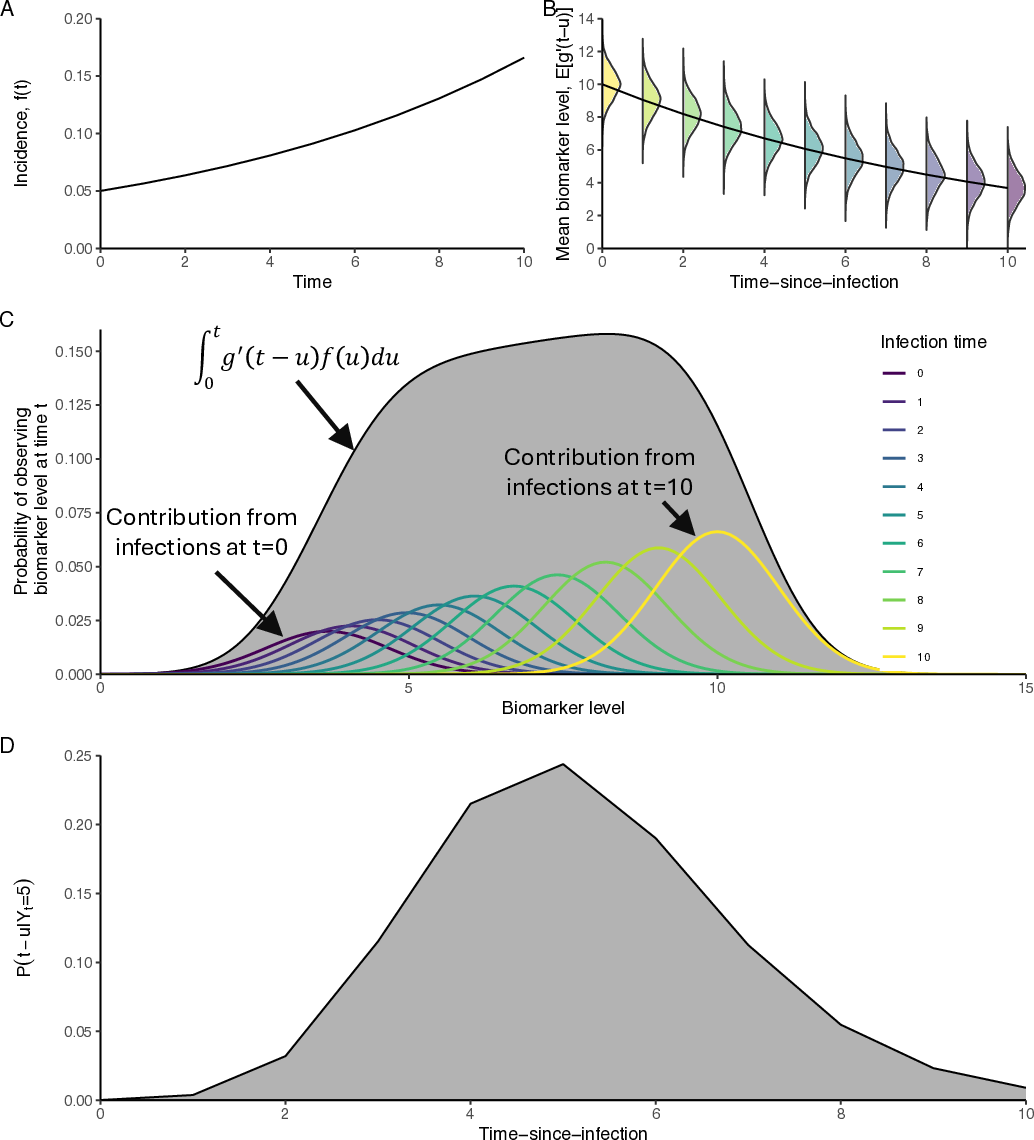
**

**Figure S4. Illustrative example of the mixture distribution of observations from infected individuals.** (A) Example incidence curve, $f\left( t \right)={0.05e}^{0.12t}$. (B) Example within-host model, $g^{'}\left( t-u \right)=Normal(10e^{-0.1\left( t-u \right)}, 1)$. (C) Observed biomarker distribution from infected individuals on day *t* given as the convolution of (A) and (B). Colored lines show the contribution from each previous day of possible infection. (D) Probability of time-since-infection given observed biomarker level $Y_{t}=5$ at $t=10$.

In practice, it is also important to account for undetectable measurements in the model, given that not all infected individuals will be detectable on the chosen assay. Similarly, if the chosen biomarker is not a serological marker of past infection but related to the current infection state (e.g., a viral load), then the latent parameter of interest is not whether an individual has ever been infected, but if they are currently infected. These possibilities are implicit in *Equation 16* above (i.e., $g^{'}$ and $h$ account for $P\left( Y_{t}=\emptyset| {t-u, Z}_{t}=1 \right)$ and $P\left( Y_{t}=\emptyset| Z_{t}=0 \right)$, where $\emptyset$ represents an undetectable or negative measurement).

For clarity, $P\left( Y_{t}=\emptyset| {t-u, Z}_{t}=1 \right)$should capture both the probability of a false negative result and the probability that the true biomarker level has declined below the limit of detection of the assay, and $P\left( Y_{t}=\emptyset| Z_{t}=0 \right)$ gives the specificity. Note that this is the same model as applied to deconvolution infection incidence from incidence of symptomatic cases when $g'$ is defined as the incubation period distribution [9,10].

- 1. **Link to time-since-infection methods**

*Equation 16*, *Equation 6,* and *Equation 9* do not explicitly represent the time-since-infection and infection state for each individual and instead integrate these latent parameters out. However, there are many use cases where the time-since-infection is itself a parameter of interest. Although in practice methods approaches to these problems can be complex to implement and require longitudinal data for each individual, they are conceptually simple and directly related to the above models.

Reintroducing the latent time-since-infection parameter, $t_{i}$into $P\left( Y_{t} \right)$ gives:

Equation 17

$$P\left( t_{i}=u | Y_{t}=y \right)=\frac{P\left( Y_{t}=y | t_{i}=u \right)P(t_{i}=u)}{P(Y_{t}=y)}$$

$$=\frac{g^{'}\left( t-u \right)f(u)}{\int_{0}^{t} g^{'}\left( t-u \right)f\left( u \right)du+h(Z_{t}=0)(1-\int_{0}^{t} f\left( u \right)du\text{)}}$$

Thus, if $g^{'}\left( t-u \right)$ and $f\left( u \right)$ are known then we can easily calculate the probability of each possible value for $t-u$ conditional on the observed biomarker level (e.g., likelihood surface in **Figure S4D**). In practice, however, these functions are not known and thus we are usually interested in jointly estimating parameters for these models alongside the unknown infection time parameters. Furthermore, when we are interested in the time-since-infection for just one infection event of an individual rather than the probability of any time-since-infection for any individual, a survival analysis framework is needed to account for the probability that an individual was infected on a given day ($f(u)\text{ }$) and not before ($1-\int_{0}^{u} f\left( v \right)dv$), as in [11,12].

Because these are population-level parameters, we are also then interested in combining data from multiple individuals and observations from multiple timepoints:

Equation 17

$$P\left( t_{1},t_{2},\ldots,t_{n}, \varphi,\theta| \boldsymbol{Y} \right)=\frac{\prod_{i} \prod_{j} P\left( Y_{i,j} | t_{i},\theta\right)P\left( t_{i} | \varphi\right)P\left( \varphi\right)P(\theta)}{\prod_{i} \prod_{j} P(Y_{i,j})}$$

$$\propto\prod_{i} \prod_{j} P\left( Y_{i,j} | t_{i},\theta\right)P\left( t_{i} | \varphi\right)P\left( \varphi\right)P(\theta)$$

Where $P\left( t_{i} | \varphi\right)$ is the probability of becoming infected at time $t_{i}$, given parameters $\varphi$ of a generic incidence model, and $P\left( Y_{i,j} | t_{i},\theta\right)$ describes the distribution of observations for individual *i* at time *j* given the time-since-infection and some model parameters. Thus, we have a generic model for estimating individual infection times, as described previously [13].

It is also common to assume that all naïve individuals are undetectable and thus $Z_{t}$ is often fixed based on some criteria for positivity. However, where infection status cannot be reliably assigned (e.g., when using assays with poor sensitivity and specificity or where antibody waning is substantial), it is instead possible to jointly estimate $Z_{t}$ alongside the time-since-infection using reversible jump Markov chain Monte Carlo [11,14], nested mixture models [15], or beta-Bernoulli processes [13]. The choice of model for $P\left( t_{i} | \varphi\right)P\left( \varphi\right)$ is not necessarily trivial, and its flexibility should be appropriate to the level of information available in the data. For example, a Gaussian Process model or flexible non-parametric function (e.g., splines) might be used, as well as simple parametric models or constants [16]. In practice, some methods rely on a two-step approach, first estimating values for the within-host model parameters $\theta$ using data from individuals with known infection times, as well as estimating individual infection times first, before estimating the population-level incidence curve [3].

**References**

1. Hens N, Aerts M, Faes C, Shkedy Z, Lejeune O, Van Damme P, et al. Seventy-five years of estimating the force of infection from current status data. Epidemiol Infect. 2010;138: 802–812.

2. Hens N, Shkedy Z, Aerts M, Faes C, Van Damme P, Beutels P. Modeling Infectious Disease Parameters Based on Serological and Social Contact Data. Springer New York, NY. 2012. https://doi.org/10.1007/978-1-4614-4072-7

3. Wilber MQ, Webb CT, Cunningham FL, Pedersen K, Wan X-F, Pepin KM. Inferring seasonal infection risk at population and regional scales from serology samples. Ecology. 2020;101: e02882.

4. Menezes A, Takahashi S, Routledge I, Metcalf CJE, Graham AL, Hay JA. serosim: An R package for simulating serological data arising from vaccination, epidemiological and antibody kinetics processes. PLoS Comput Biol. 2023;19: e1011384.

5. Andraud M, Lejeune O, Musoro JZ, Ogunjimi B, Beutels P, Hens N. Living on Three Time Scales: The Dynamics of Plasma Cell and Antibody Populations Illustrated for Hepatitis A Virus. Fraser C, editor. PLoS Comput Biol. 2012;8: e1002418.

6. Brookmeyer R. Measuring the HIV/AIDS Epidemic: Approaches and Challenges. Epidemiol Rev. 2010;32: 26–37.

7. Grenfell BT, Anderson RM. The estimation of age-related rates of infection from case notifications and serological data. J Hyg . 1985;95: 419–436.

8. Gay NJ, Vyse AJ, Enquselassie F, Nigatu W, Nokes DJ. Improving sensitivity of oral fluid testing in IgG prevalence studies: application of mixture models to a rubella antibody survey. Epidemiol Infect. 2003;130: 285–291.

9. Miller AC, Hannah LA, Futoma J, Foti NJ, Fox EB, D’Amour A, et al. Statistical Deconvolution for Inference of Infection Time Series. Epidemiology. 2022;33: 470–479.

10. Brookmeyer R. Reconstruction and future trends of the AIDS epidemic in the United States. Science. 1991;253: 37–42.

11. Salje H, Cummings DAT, Rodriguez-Barraquer I, Katzelnick LC, Lessler J, Klungthong C, et al. Reconstruction of antibody dynamics and infection histories to evaluate dengue risk. Nature. 2018. pp. 719–723. doi:10.1038/s41586-018-0157-4

12. Salje H, Alera MT, Chua MN, Hunsawong T, Ellison D, Srikiatkhachorn A, et al. Evaluation of the extended efficacy of the Dengvaxia vaccine against symptomatic and subclinical dengue infection. Nat Med. 2021;27: 1395–1400.

13. Hay JA, Minter A, Ainslie KEC, Lessler J, Yang B, Cummings DAT, et al. An open source tool to infer epidemiological and immunological dynamics from serological data: Serosolver. PLoS Comput Biol. 2020;16: e1007840.

14. Tsang TK, Perera RAPM, Fang VJ, Wong JY, Shiu EY, So HC, et al. Reconstructing antibody dynamics to estimate the risk of influenza virus infection. Nat Commun. 2022;13: 1557.

15. Bollaerts K, Aerts M, Shkedy Z, Faes C, Van der Stede Y, Beutels P, et al. Estimating the population prevalence and force of infection directly from antibody titres. Stat Modelling. 2012;12: 441–462.

16. Hozé N, Paireau J, Lapidus N, Tran Kiem C, Salje H, Severi G, et al. Monitoring the proportion of the population infected by SARS-CoV-2 using age-stratified hospitalisation and serological data: a modelling study. Lancet Public Health. 2021;6: e408–e415.
